# Effectiveness of nutrition education program and omega-3 supplementation on body weight, liver enzyme, lipid profile among non-alcoholic fatty liver disease patients

**DOI:** 10.1186/s40795-026-01309-0

**Published:** 2026-04-16

**Authors:** Dilkhosh Shamal Ramadhan, Ibrahim Hasan Mustafa

**Affiliations:** Department of Community, College of Nursing, Hawler Medical University, Erbil, Iraq

**Keywords:** Non-alcoholic fatty liver disease, Omega-3 fatty acids, Nutrition education, Liver enzymes, Lipid profile, Erbil city

## Abstract

**Background and aim:**

Non-alcoholic fatty liver disease (NAFLD) represents a growing metabolic and hepatic health challenge worldwide, particularly in low- and middle-income regions such as Iraq. This study aimed to evaluate the safety and effectiveness of a combined nutrition education program and omega-3 fatty acid supplementation in improving anthropometric, biochemical, and hepatic outcomes among patients with NAFLD in Erbil City.

**Method:**

This randomized controlled trial study was conducted from November 3rd, 2024 to March 3rd, 2025, at the outpatient clinic of Rizgary Teaching Hospital in Erbil City, Kurdistan Region, Iraq, using systematic sampling method. Data were collected using a structured questionnaire consisting of sociodemographic and clinical characteristics, anthropometric and biochemical measurements, the International Physical Activity Questionnaire (IPAQ), and dietary behavior scales. Statistical analyses were performed using IBM SPSS version 26 (IBM Corp., Armonk, NY).

**Results:**

A total of 162 patients with non-alcoholic fatty liver disease (NAFLD) were included in the study. Both omega-3 supplementation and nutrition education interventions significantly improved anthropometric and biochemical parameters compared to baseline values. The nutrition education group achieved greater reductions in body weight (− 2.83 kg, *p* < 0.01), BMI (− 1.00 kg/m^2^, *p* < 0.01), and liver enzymes (ALT and AST, *p* < 0.01), along with improved lipid profiles and dietary behaviors. Total physical activity levels increased significantly in both intervention groups (*p* < 0.001). Hierarchical multiple regression identified nutrition education (β = 0.428, *p* < 0.001), omega-3 supplementation (β = 0.312, *p* < 0.001), physical activity change (β = 0.284, *p* < 0.001), and compliance (β = 0.258, *p* = 0.001) as key predictors of treatment success, collectively explaining 61% of the variance in outcomes.

**Conclusions:**

The study demonstrated that a nutrition education and omega-3 supplementation program effectively improved hepatic function, lipid profile, and physical activity among patients with NAFLD in Erbil City. Integrating such low-cost, behaviorally oriented interventions into clinical practice may provide a sustainable approach for NAFLD management in resource-limited settings.

**Trial registration:**

The clinical trial was prospectively registered at ClinicalTrials.gov (Identifier: NCT06627114; Registration date: October 2, 2024).

**Supplementary Information:**

The online version contains supplementary material available at 10.1186/s40795-026-01309-0.

## Introduction

Non-alcoholic fatty liver disease (NAFLD) is a major global health burden, the prevalence and outcomes of which have long been underestimated and underaddressed. Inadequate screening tools and limited population-based studies have resulted in wide-ranging global estimates of 25% to 30% of adults living with fatty liver disease [1, 2]. Although NAFLD affects populations worldwide, it disproportionately impacts individuals in low- and middle-income regions, where obesity and metabolic syndrome are rapidly increasing due to urbanization and poor dietary habits. Recognizing this epidemiological transition, WHO and related agencies have prioritized metabolic liver disorders as part of the 2021–2030 global action framework on noncommunicable diseases [3, 4]. Nonetheless, NAFLD remains underrecognized in national health strategies, earning its reputation as “the silent epidemic of metabolic dysfunction. Recent data indicate that NAFLD now affects over 1 billion individuals globally, making it the most common cause of chronic liver disease worldwide. The Middle East and South America record the highest regional prevalence rates (32–35%), followed by Asia (27%), Europe (23%), and North America (24%) [5, 6]. Furthermore, projections suggest that by 2030, up to 50% of adults with obesity or type 2 diabetes will exhibit hepatic steatosis, highlighting the accelerating metabolic burden.

Unlike viral or alcoholic liver diseases, NAFLD is characterized by excessive hepatic fat accumulation unrelated to alcohol intake, which can progress to non-alcoholic steatohepatitis (NASH) and cirrhosis if untreated. Most affected individuals are asymptomatic or present with mild fatigue, abdominal discomfort, and metabolic abnormalities such as dyslipidemia, insulin resistance, or hypertension [7]. In contrast, those with advanced fibrosis or coexisting metabolic disorders face heightened risks of hepatic inflammation, cardiovascular events, and liver-related mortality [8, 9]. Vulnerable populations—particularly those with obesity, type 2 diabetes, or sedentary lifestyles—are at increased risk of rapid disease progression, which can lead to end-stage liver failure and hepatocellular carcinoma [10, 11]. Without effective intervention, advanced NASH carries a five-year mortality rate exceeding 40%, underscoring the urgency of addressing metabolic liver health through preventive and therapeutic strategies [12]. Globally, NAFLD is now the second leading indication for liver transplantation after viral hepatitis, and its associated cardiovascular risk surpasses that of many cancers [13]. Studies show that up to 70% of individuals with type 2 diabetes have concurrent NAFLD, while 25% progress to NASH with significant fibrosis [14].

Current clinical management of NAFLD focuses primarily on lifestyle modification, including calorie restriction, physical activity, and dietary balance, which remain the cornerstone of therapy worldwide. Pharmacological options such as pioglitazone and vitamin E have shown modest benefits but are limited by adverse effects and inconsistent long-term efficacy [15]. Despite these challenges, sustained dietary modification remains difficult, and adherence rates to long-term lifestyle change are low among high-risk individuals, especially in resource-limited settings. Furthermore, the rising prevalence of obesity and type 2 diabetes magnifies the urgency of developing adjunct nutritional and pharmacological therapies that can effectively reduce hepatic fat and improve metabolic biomarkers. Emerging evidence suggests that omega-3 fatty acids and structured nutrition education may jointly modulate lipid metabolism and hepatic steatosis, providing dual benefit in disease management [16, 17]. These integrative interventions represent a progressive approach to NAFLD therapy by targeting both behavioral and biochemical determinants of liver health. Nevertheless, variations in dosing, adherence, and long-term sustainability continue to limit clinical translation and large-scale implementation. Clinical trials conducted in Japan, Italy, and the United States have reported that omega-3 supplementation (2–4 g/day) can lower hepatic triglyceride content by up to 35% and reduce ALT and AST by 25% within 12 weeks [18, 19]. Likewise, nutrition education interventions emphasizing the Mediterranean or DASH diet have achieved significant improvements in insulin sensitivity and lipid profiles [20]. However, a major challenge persists in maintaining adherence beyond six months, especially among younger adults with inconsistent dietary behaviors.

Given the high global burden of NAFLD and the absence of approved pharmacotherapy, there is an urgent need for effective, low-cost, and accessible lifestyle-based interventions. Omega-3 fatty acids have demonstrated anti-inflammatory and lipid-lowering effects in metabolic diseases, while nutrition education programs enhance self-regulation and dietary compliance among affected individuals [10]. Previous clinical trials have reported improvements in hepatic enzymes, triglycerides, and weight reduction following combined lifestyle and omega-3 interventions in NAFLD patients [21]. The aim, therefore, of this study was to evaluate the safety and effectiveness of a combined nutrition education and omega-3 supplementation program on metabolic and hepatic outcomes in NAFLD patients.

## Research question

What is the effectiveness of a nutrition education program and omega-3 fatty acid supplementation in improving body weight, liver enzyme levels, and lipid profile among patients with non-alcoholic fatty liver disease?

## Methods

### Study design, setting, period, and sampling

**Fig. 1:**
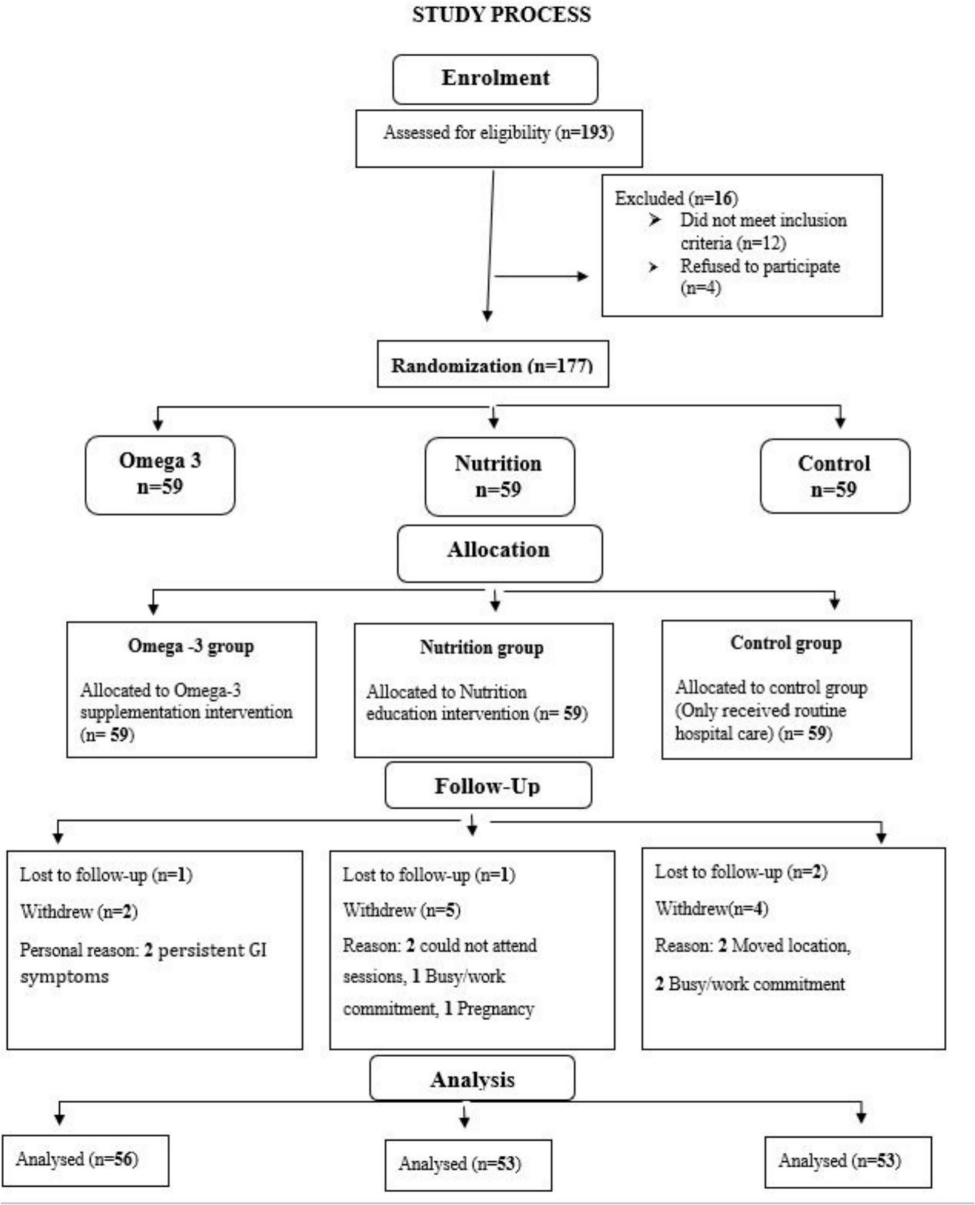
CONSORT flow diagram of participant enrollment, allocation, follow-up, and analysis in the omega-3, nutrition education, and control groups

This study was a randomized controlled trial study conducted at the outpatient clinic of Rizgary Teaching Hospital in Erbil City, Kurdistan Region, Iraq. Data were collected from November 3rd, 2024 to March 3rd, 2025, using a systematic sampling method. This randomized controlled trial was designed, conducted, and reported in accordance with the CONSORT 2025 guidelines, and the completed CONSORT checklist is provided as Supplementary File 1. The participant flow through each stage of the trial is shown in Fig. 1.

### Randomization, allocation concealment, and blinding

The Simple randomization was used to allocate participants to either the interventions or control group. The randomization sequence was generated independently by a biostatistician using the using Microsoft Excel program, ensuring that each participant had an equal probability of assignment to either group. Allocation concealment was maintained by securely storing the randomization codes in a locked safe, accessible only to the researcher until completion of data entry. This procedure prevented advance knowledge of group assignment and minimized the risk of selection bias. Participants were aware of the intervention they received; however, they were not informed about the total number of study groups or which intervention was considered experimental. This approach represented partial participant blinding and was implemented to reduce expectation and contamination bias. Outcome assessors, including laboratory personnel and the radiologist, were blinded to participants’ group allocation. All outcome measurements were conducted using standardized and objective procedures across all participants to minimize the risk of measurement and detection bias.

### Sample size

The total sample for this study comprised 162 patients diagnosed with non-alcoholic fatty liver disease (NAFLD) who met the inclusion criteria. The initial sample size was determined using G*Power version 3.1.9.4, assuming an effect size of 0.25, an α error of 0.05, and a statistical power of 80%, yielding a target of 159 participants. To compensate for potential attrition, the sample was increased to 162 participants.

### Inclusion/exclusion

The inclusion criteria were patients aged 18 years and above, diagnosed with grade 1 or 2 NAFLD confirmed by ultrasound, with BMI ≥ 25 kg/m^2^, and who were able to use a mobile phone for communication. Exclusion criteria included pregnancy or breastfeeding, use of omega-3 or PUFA supplements in the previous six months, chronic liver disease of other causes (autoimmune hepatitis, Wilson’s disease, etc.), hormone therapy, or use of hepatotoxic drugs (e.g., amiodarone, tamoxifen, methotrexate) within three months prior to the study.

### Study tools and data collection

The questionnaire was divided into four main parts. The first part collected sociodemographic and clinical information about participants, including year of birth, gender, marital status, religion, level of education, occupation, nature of work, residential area, family income, past medical history, current medication, supplement use, smoking status, and food allergies. The second part recorded anthropometric and biochemical parameters, such as height, weight, body mass index (BMI), waist and hip circumference, waist-to-hip ratio, lipid profile (triglycerides, total cholesterol, LDL, HDL), liver enzyme levels (AST, ALT, ALP), and liver ultrasound grade at baseline and after the 12-week intervention. The third part consisted of the International Physical Activity Questionnaire (IPAQ), which measured the duration and frequency of walking, moderate, and vigorous physical activities in minutes per week to estimate total metabolic equivalent (MET)-minutes. The fourth part assessed dietary behaviors using two subscales: the Dietary Habits Scale (12 items rated on a 5-point frequency scale from “never” to “always”) and the Dietary Pattern Questionnaire, a short food-frequency tool evaluating the weekly or monthly consumption of food groups such as meats, grains, dairy products, vegetables, fruits, fats, sweets, legumes, and beverages. The questionnaire was originally developed in English and translated into Kurdish using the forward–backward translation method to ensure conceptual equivalence and linguistic accuracy. Data were collected through face-to-face interviews with participants who met the inclusion criteria, and each participant was allotted approximately 20–25 min to complete the questionnaire.

### Pilot study

The study questionnaire was pilot-tested with 18 patients diagnosed with non-alcoholic fatty liver disease (NAFLD) between October 1st-30th, 2024, and October 30th, 2025. The aim of the pilot was to assess the clarity, internal consistency, and overall reliability of the questionnaire items prior to their use in the main study. Reliability analysis was performed using Cronbach’s alpha. The International Physical Activity Questionnaire (IPAQ) demonstrated an excellent internal consistency with a Cronbach’s alpha of 0.95 [22]. Similarly, the Dietary Habits Scale showed a strong reliability with an overall Cronbach’s alpha of 0.84. The pilot testing also confirmed the feasibility of the study procedures and the comprehensibility of all data collection tools. Importantly, data obtained from the pilot phase were excluded from the final analysis.

### Measures

A structured questionnaire was specifically developed by the research team for the purposes of this study. The English version of the instrument is provided as Supplementary File 2.

#### Sociodemographic characteristics

The first section of the questionnaire included sociodemographic information of patients such as year of birth, gender, marital status, religion, level of education, occupation, nature of work, residential area, family income, past medical history, medication use, supplement intake, smoking status, and food allergies.

#### Anthropometric and biochemical measurement

The second section focused on anthropometric and biochemical measurements, which included height, weight, body mass index (BMI), waist circumference, hip circumference, and waist-to-hip ratio measured at both baseline and endline. Biochemical parameters included lipid profile (triglycerides, total cholesterol, HDL, LDL) and liver enzymes (AST, ALT, ALP). These parameters were measured at baseline and after the 12-week intervention to evaluate treatment effectiveness.

#### International Physical Activity Questionnaire (IPAQ)

The third section utilized the International Physical Activity Questionnaire (IPAQ) [23], a standardized instrument designed to assess total physical activity levels through three components: walking, moderate activity, and vigorous activity. Participants reported the frequency (days per week) and duration (minutes per day) for each activity type, and results were converted into MET-minutes per week to estimate overall physical activity levels. The Cronbach’s alpha for the IPAQ was 0.95, indicating excellent reliability in the current study.

#### Dietary habits and dietary pattern questionnaires

The Dietary Habits Scale 12 items measured frequency of unhealthy eating behaviors on a five-point Likert scale. Lower scores indicate healthier dietary habits [24], snacking, and fast-food consumption on a five-point Likert scale ranging from never 0 to always 5. The Dietary Pattern Questionnaire was a short food frequency survey evaluating the frequency of consumption of various food groups including meats, grains, dairy, vegetables, fruits, fats, legumes, sweets, and beverages [23]. Higher scores indicated healthier dietary patterns. Both tools demonstrated acceptable validity and reliability and were used to capture lifestyle and nutritional changes among patients with NAFLD.

### Ethical approval and inform consent

This study was conducted in accordance with the ethical principles outlined in the Declaration of Helsinki (2013 revision) and followed national and institutional research ethics standards. Ethical approval for the study was obtained from the Ethical Committee of the College of Nursing, Hawler Medical University, under approval code 243, granted on June 2, 2024. Additional permissions were obtained from the Directorate of Health–Erbil and the Rizgary Teaching Hospital prior to data collection. The clinical trial was prospectively registered at ClinicalTrials.gov with the identifier NCT06627114 (Release Date: October 2, 2024). All participants were provided with detailed information regarding the study’s objectives, procedures, potential risks, and benefits. Written informed consent was obtained from each participant prior to enrollment. Participation was entirely voluntary, and participants were assured of their right to withdraw at any stage without penalty, as well as the confidentiality and anonymity of their personal data.

### Intervention procedures

Patients randomized to the nutrition education program participated in structured sessions grounded in the Theory of Planned Behavior (TPB). The intervention consisted of three interactive sessions, each lasting 45–60 min, conducted in small groups to promote engagement and peer learning. Each session combined lectures, discussions, and visual demonstrations to enhance comprehension and retention. Session 1 focused on understanding non-alcoholic fatty liver disease (NAFLD), its causes, risk factors, and the role of nutrition in disease prevention. Session 2 emphasized healthy dietary practices, including portion control, the importance of fruits, vegetables, complex carbohydrates, low-fat dairy products, healthy fats, white meats, and fish, while discouraging the intake of refined carbohydrates and foods high in saturated fats. The educational materials were culturally adapted to reflect common Kurdish dietary practices and locally available foods in Erbil. Participants were encouraged to modify traditional meals by reducing the consumption of fried foods, sweets, and refined carbohydrates while increasing intake of vegetables, fruits, legumes, whole grains, and fish that are commonly accessible in local markets. Practical examples based on typical Kurdish meals such as rice-based dishes, bread consumption, and meat preparation methods were used during the sessions to improve understanding and facilitate adherence to healthier dietary patterns.

Session 3 addressed behavioral modification strategies, such as goal setting, self-monitoring, and overcoming barriers to adherence. To reinforce learning, weekly WhatsApp or SMS reminders were sent throughout the study period, encouraging participants to maintain dietary and lifestyle changes. The intervention’s design was based on TPB’s constructs—attitude, subjective norms, and perceived behavioral control—to improve intention and adherence to healthier eating behaviors [25].

Patients assigned to the omega-3 supplementation group received a molecularly distilled fish oil supplement (Oxford Vitality, The Oxford Health Company Ltd., Bicester, United Kingdom). Each softgel capsule contained 1,000 mg of purified fish oil, providing approximately 550 mg of total omega-3 fatty acids, including 330 mg of eicosapentaenoic acid (EPA) and 220 mg of docosahexaenoic acid (DHA), along with 3.4 mg of vitamin E (D-alpha-tocopherol) to maintain stability. Participants were instructed to consume two softgel capsules daily, preferably with meals (morning and evening), for 12 consecutive weeks. The dosage and duration were selected according to prior clinical trials demonstrating the beneficial effects of omega-3 fatty acids on hepatic steatosis, lipid profile, and liver enzymes among patients with NAFLD [26]. Adherence to omega-3 supplementation was monitored through regular follow-up communication and participant self-report during scheduled visits and weekly reminder messages throughout the intervention period.

### Statistical analysis

Data were summarized and reported using frequency and percentage for qualitative variables such as sociodemographic and clinical characteristics (e.g., age group, gender, education level, and comorbidities), while quantitative variables were expressed as mean and standard deviation for parameters including body weight, BMI, waist circumference, liver enzyme levels, lipid profile, and physical activity (MET-min/week). Group comparisons were analyzed using Chi-square tests for categorical variables and independent t-tests or one-way ANOVA for continuous variables, followed by Tukey’s HSD post hoc test for pairwise differences. Paired t-tests assessed within-group changes between baseline and endline measurements, and Pearson’s correlation coefficient examined relationships between dietary habits, dietary patterns, and demographic or clinical variables. To identify predictors of treatment response, hierarchical multiple linear regression was conducted in three models: Model 1 included demographic predictors (age, gender, marital status, education, and income); Model 2 added baseline clinical and biochemical factors (weight, ALT, AST, triglycerides, and LDL-C); and Model 3 incorporated intervention-related variables (nutrition education, omega-3 supplementation, compliance, physical activity change, and dietary pattern change). Model fit was evaluated using the Durbin–Watson test, Shapiro–Wilk test, and Levene’s test, while multicollinearity was checked using the variance inflation factor (VIF < 3). All analyses were performed using IBM SPSS Statistics version 26.0 (IBM Corp., Armonk, NY), and a p-value < 0.05 was considered statistically significant.

## Results

### Socio-demographic characteristics of participants

A total of 162 patients with non-alcoholic fatty liver disease (NAFLD) participated in the study, equally distributed among the control, omega-3, and nutrition education groups. The overall mean age ranged between 46.06 ± 10.88 years in the control group, 49.82 ± 8.92 years in the omega-3 group, and 46.98 ± 11.54 years in the nutrition group, indicating that most participants were middle-aged adults. The majority were aged 38–56 years (64.2%, 71.4%, and 64.2% respectively), while females slightly outnumbered males in all groups. Most participants were married (88.7–92.5%) and Muslim (> 98%). Significant group differences were found for educational level (*p* < 0.01), occupation (*p* = 0.02), and residential area (*p* < 0.01). Participants in the nutrition group had higher proportions of primary and diploma education, while those in the omega-3 group were more frequently employed and nearly all resided in urban areas. Across all groups, most patients reported moderate income levels (500,000–1,000,000 IQD, about 50%) and no major medical history or medication use, while the majority were non-smokers (over 88%) and had no food allergies. These baseline differences were noted and considered in subsequent statistical analyses. Detailed demographics and other variables are presented in Table 1.

**Table 1:** Socio-Demographic Characteristics of Non-Alcoholic Fatty Liver Disease (NAFLD) Patients by Study Groups (*N* = 162)

**Variable**	**Type of Variable**	**Group Classification**			
		**Control Group F (%)**	**Omega-3 Group F (%)**	**Nutrition Group F (%)**	***p*-value**
Age Group (years)	19–37	12 (22.6)	5 (8.9)	9 (17.0)	0.37
	38–56	34 (64.2)	40 (71.4)	34 (64.2)	
	57–76	7 (13.2)	11 (19.6)	10 (18.9)	
	Mean ± SD	46.06 ± 10.88	49.82 ± 8.92	46.98 ± 11.54	
Gender	Male	25 (47.2)	20 (35.7)	30 (56.6)	0.09
	Female	28 (52.8)	36 (64.3)	23 (43.4)	
Marital Status	Single	4 (7.5)	6 (10.7)	4 (7.5)	0.37
	Married	47 (88.7)	49 (87.5)	49 (92.5)	
	Widowed	0 (0.0)	1 (1.8)	0 (0.0)	
	Divorced	2 (3.8)	0 (0.0)	0 (0.0)	
Religion	Muslim	53 (100.0)	55 (98.2)	52 (98.1)	0.41
	Christian	0 (0.0)	0 (0.0)	1 (1.9)	
	Yezidi	0 (0.0)	1 (1.8)	0 (0.0)	
Level of Education	No formal education	10 (18.9)	5 (8.9)	2 (3.8)	<.01
	Able to read/write	13 (24.5)	3 (5.4)	2 (3.8)	
	Primary School	10 (18.9)	8 (14.3)	20 (37.7)	
	Intermediate School	3 (5.7)	11 (19.6)	8 (15.1)	
	Secondary School	3 (5.7)	8 (14.3)	0 (0.0)	
	Diploma	5 (9.4)	8 (14.3)	15 (28.3)	
	Bachelor’s Degree	7 (13.2)	11 (19.6)	6 (11.3)	
	Postgraduate	2 (3.8)	2 (3.6)	0 (0.0)	
Occupation	Unemployed	1 (1.9)	0 (0.0)	2 (3.8)	0.02
	Employee	15 (28.3)	30 (53.6)	22 (41.5)	
	Student	2 (3.8)	0 (0.0)	0 (0.0)	
	Housewife	22 (41.5)	20 (35.7)	16 (30.2)	
	Retired	1 (1.9)	0 (0.0)	0 (0.0)	
	Self-Business	0 (0.0)	1 (1.8)	5 (9.4)	
	Worker/Handworker	12 (22.6)	5 (8.9)	8 (15.1)	
Residential Area	Urban	40 (75.5)	55 (98.2)	46 (86.8)	<.01
	Suburban	13 (24.5)	1 (1.8)	7 (13.2)	
Family Income (IQD)	≤ 500,000	11 (20.8)	4 (7.1)	5 (9.4)	0.30
	500,000–1,000,000	26 (49.1)	30 (53.6)	30 (56.6)	
	≥ 1,000,000	16 (30.2)	21 (37.5)	18 (34.0)	
Past Medical History	None	32 (60.4)	30 (53.6)	34 (64.2)	0.20
	Hypertension	12 (22.6)	14 (25.0)	12 (22.6)	
	Diabetes Mellitus	2 (3.8)	4 (7.1)	5 (9.4)	
	Both HTN and DM	5 (9.4)	4 (7.1)	1 (1.9)	
	Others	2 (3.8)	0 (0.0)	0 (0.0)	
Medication in Use	None	34 (64.2)	31 (55.4)	36 (67.9)	0.69
	Metformin	3 (5.7)	5 (8.9)	5 (9.4)	
	Antihypertensive	11 (20.8)	14 (25.0)	10 (18.9)	
	Combined Therapy	3 (5.7)	4 (7.1)	1 (1.9)	
Supplement in Use	Yes	7 (13.2)	7 (12.5)	4 (7.5)	0.60
	No	46 (86.8)	49 (87.5)	49 (92.5)	
Smoking Status	Yes	6 (11.3)	5 (8.9)	5 (9.4)	0.91
	No	47 (88.7)	51 (91.1)	48 (90.6)	
Ex-Smoker	Yes	0 (0.0)	1 (1.8)	0 (0.0)	0.39
	No	53 (100.0)	55 (98.2)	53 (100.0)	
Food Allergy	Yes	0 (0.0)	1 (1.8)	0 (0.0)	0.39
	No	53 (100.0)	55 (98.2)	53 (100.0)	

Data analyzed using Chi-square test; values represent frequency (percentage)

*p* <.05 considered statistically significant

### Changes in anthropometric, biochemical, and hepatic parameters within groups

Participants in the omega-3 and nutrition education groups showed statistically significant changes in several anthropometric and biochemical parameters compared with baseline values. Mean body weight decreased from 86.63 ± 11.80 to 85.83 ± 11.98 kg in the omega-3 group and from 90.07 ± 12.91 to 87.24 ± 13.05 kg in the nutrition group, with corresponding reductions in BMI from 32.36 ± 4.78 to 32.02 ± 4.88 kg/m^2^ and from 32.27 ± 4.88 to 31.27 ± 4.93 kg/m^2^ (both *p* < 0.01). Improvements in waist circumference were also observed in the nutrition group (*p* < 0.01). Lipid profile analyses indicated significant reductions in triglycerides, total cholesterol, and LDL-C, alongside increased HDL-C levels in the nutrition group (*p* < 0.01). Liver enzyme levels, including AST and ALT, decreased significantly in both omega-3 and nutrition groups, while ALP levels declined only in the nutrition group (*p* < 0.01). Post-intervention liver ultrasound findings showed that the proportion of patients with normal liver echogenicity increased in the omega-3 group (12.5%) and the nutrition group (28.3%) compared with the control group (5.7%), with statistically significant differences observed between groups (χ^2^ = 10.21, *p* < 0.01). For further details, see Table 2.

**Table 2:** Comparison of anthropometric, biochemical, and liver ultrasound parameters at baseline and end among NAFLD Patients (*N* = 162)

Parameter	Control (*n* = 53)	Omega-3 (*n* = 56)	Nutrition Education (*n* = 53)	Within-Group Significance
Anthropometric Measurements				
Weight (kg)	85.59 ± 14.34 → 86.50 ± 14.59	86.63 ± 11.80 → 85.83 ± 11.98	90.07 ± 12.91 → 87.24 ± 13.05	↓ *Sig* in Ω−3 & Nutrition (*p* <.01)
BMI (kg/m^2^)	32.29 ± 7.15 → 31.48 ± 3.85	32.36 ± 4.78 → 32.02 ± 4.88	32.27 ± 4.88 → 31.27 ± 4.93	↓ *Sig* in Ω−3 & Nutrition (*p* <.01)
Waist circumference (cm)	96.51 ± 7.43 → 97.42 ± 7.88	98.83 ± 6.90 97.58 ± 7.12	98.72 ± 8.15 → 95.75 ± 8.25	↓ *Sig* in Nutrition (*p* <.01)
Biochemical Parameters – Lipid Profile				
Triglycerides (mg/dL)	192.06 ± 41.53 → 198.61 ± 42.89	209.25 ± 65.16 → 184.63 ± 54.74	208.22 ± 48.11 → 182.34 ± 44.67	↓ *Sig* in Ω−3 & Nutrition (*p* <.01)
Total Cholesterol (mg/dL)	218.27 ± 36.04 → 223.32 ± 41.71	227.33 ± 50.74 → 217.19 ± 49.73	230.01 ± 43.46 → 205.09 ± 36.93	↓ *Sig* in Ω−3 & Nutrition (*p* <.01)
LDL-C (mg/dL)	103.32 ± 21.15 → 112.05 ± 26.61	96.33 ± 23.66 → 96.99 ± 21.87	104.49 ± 23.94 → 89.79 ± 20.24	↓ *Sig* in Nutrition (*p* <.01)
HDL-C (mg/dL)	38.02 ± 9.17 → 37.31 ± 7.91	37.73 ± 17.99 → 41.93 ± 9.85	35.88 ± 5.85 → 40.55 ± 5.51	↑ *Sig* in Nutrition (*p* <.01)
Biochemical Parameters – Liver Enzymes				
AST (U/L)	27.46 ± 6.92 → 28.91 ± 7.36	29.93 ± 8.42 → 25.76 ± 7.79	30.43 ± 8.38 → 24.58 ± 5.94	↓ *Sig* in Ω−3 & Nutrition (*p* <.01)
ALT (U/L)	29.36 ± 6.73 → 31.47 ± 8.44	33.60 ± 11.75 → 27.21 ± 9.23	33.49 ± 10.40 → 25.78 ± 7.41	↓ *Sig* in Ω−3 & Nutrition (*p* <.01)
ALP (U/L)	81.35 ± 15.57 → 84.68 ± 18.44	88.82 ± 28.33 → 95.19 ± 148.76	86.68 ± 15.60 → 72.07 ± 10.07	↓ *Sig* in Nutrition (*p* <.01)
Liver Ultrasound Grade (End of Study)	Grade I = 37 (69.8%)Grade II = 13 (24.5%)Normal = 3 (5.7%)	Grade I = 45 (80.4%)Grade II = 4 (7.1%)Normal = 7 (12.5%)	Grade I = 35 (66.0%)Grade II = 3 (5.7%)Normal = 15 (28.3%)	*χ*^*2*^ = 10.21, *p* <.01

Data are presented as mean ± standard deviation (SD) for continuous variables and frequency (%) for categorical variables. Within-group comparisons were analyzed using paired t-tests; between-group differences assessed by one-way ANOVA (continuous) and Chi-square test (categorical). Significance level set at *p* <.05. Downward (↓) or upward (↑) arrows denote direction of improvement

### Total physical activity levels among NAFLD patients by study group

Differences in total physical activity levels were observed among the three study groups. Participants in the nutrition education group had the highest mean physical activity level (2,595.02 ± 695.88 MET-min/week), followed by the omega-3 supplementation group (2,430.86 ± 545.35 MET-min/week), while the control group recorded the lowest mean value (1,698.01 ± 410.38 MET-min/week). The overall mean for all participants was 2,244.81 ± 680.61 MET-min/week, indicating a moderate level of activity across the cohort. Confidence intervals indicated limited overlap between the control and intervention groups. The observed activity range (918.15–4,200.00 MET-min/week) suggests variability in lifestyle behaviors among participants, with higher activity levels reported in the intervention groups (Table 3).

**Table 3:** Descriptive Statistics of Total Physical Activity (MET-min/week) Among NAFLD Patients by Study Group (*N* = 162)

Study Group	N	Mean (MET-min/week)	Standard Deviation (SD)	Standard Error (SE)	95% Confidence Interval for Mean	Minimum	Maximum
Control	53	**1,698.01**	410.38	56.37	1,584.90–1,811.13	918.15	2,633.53
Omega-3 Supplementation	56	**2,430.86**	545.35	72.88	2,284.81–2,576.90	1,000.00	3,920.33
Nutrition Education	53	**2,595.02**	695.88	95.59	2,403.22–2,786.83	1,256.86	4,200.00
Total (All Participants)	**162**	**2,244.81**	680.61	53.47	2,139.21–2,350.41	918.15	4,200.00

Data are presented as mean ± standard deviation (SD). Values represent total physical activity expressed in MET-minutes per week among patients with non-alcoholic fatty liver disease (NAFLD). Higher mean values indicate greater overall physical activity. Descriptive statistics include group sample size, standard error, 95% confidence interval, and observed range. Bold values represent the highest mean values among the study groups

### Comparison of total physical activity among study groups

One-way ANOVA demonstrated a statistically significant difference in total physical activity levels among the three study groups (F = 38.39, p < 0.001). The Tukey HSD post hoc test indicated that both the omega-3 supplementation group and the nutrition education group had significantly higher mean physical activity levels compared with the control group, with mean differences of − 732.84 MET-min/week and − 897.01 MET-min/week, respectively (both *p* < 0.001). However, the difference between the omega-3 and nutrition education groups was not statistically significant (*p* = 0.283). For more details, refer to Table 4.

**Table 4:** One-Way ANOVA and Tukey HSD Post Hoc Comparison of Total Physical Activity (MET-min/week) Among Study Groups (*N* = 162)

Section	Source/Comparison	Sum of Squares (SS)	df	Mean Square (MS)	F-value/Mean Difference	Standard Error	*p*-value (Sig.)	95% Confidence Interval (Lower–Upper)
A. ANOVA Results	Between Groups	24,284,944.51	2	12,142,472.25	**F = 38.39**	—	** < 0.001**	—
	Within Groups	50,295,278.09	159	316,322.50	—	—	—	—
	Total	74,580,222.60	161	—	—	—	—	—
B. Tukey HSD Post Hoc Comparisons	Control vs. Omega-3	—	—	—	**−732.84**	107.78	** < 0.001**	−987.84 – −477.84
	Control vs. Nutrition	—	—	—	**−897.01**	109.26	** < 0.001**	−1,155.49 – −638.53
	Omega-3 vs. Nutrition	—	—	—	**−164.17**	107.78	0.283	−419.17 – 90.83

One-way analysis of variance (ANOVA) was used to compare total MET-minutes per week among the three study groups (Control, Omega-3, and Nutrition Education). The F-test evaluated overall group differences, followed by Tukey HSD post hoc tests for pairwise comparisons. Mean differences represent the difference in average MET-minutes between groups. Positive values indicate higher mean activity in the first-listed group. A probability value of *p* <.01 denotes a statistically significant difference

### Comparison of baseline and endline dietary habits and patterns

Changes in dietary habits and dietary patterns were observed among participants receiving omega-3 supplementation and nutrition education, whereas no statistically significant changes were detected in the control group. In the nutrition education group, mean dietary habit scores changed from 41.77 ± 4.53 at baseline to 35.55 ± 3.68 at endline (t = 11.09, *p* < 0.001), while dietary pattern scores changed from 99.19 ± 10.38 to 103.58 ± 9.70 (t = − 3.94, *p* < 0.001). The omega-3 group also demonstrated statistically significant changes in dietary habits (*p* = 0.011) and dietary pattern scores (*p* = 0.038). In contrast, the control group showed no statistically significant changes in either measure (*p* > 0.05) (Table 5).

**Table 5:** Comparison of baseline and endline mean scores of dietary habits and dietary pattern by study group (Paired Samples t-test) (*N* = 162)

Group	Variable	Baseline Mean ± SD	Endline Mean ± SD	Mean Difference	t	df	*p*-value	Significance
Control (*n* = 53)	Dietary Habits	40.57 ± 4.24	40.28 ± 4.02	+ 0.28 ± 2.71	0.76	52	.451	NS
	Dietary Pattern	103.19 ± 14.52	103.36 ± 13.66	–0.17 ± 10.38	–0.12	52	.906	NS
Omega-3 (*n* = 56)	Dietary Habits	40.52 ± 4.90	38.32 ± 5.77	+ 2.20 ± 6.23	2.64	55	.011	**Significant** (*p* < *.05*)
	Dietary Pattern	101.84 ± 11.33	100.48 ± 10.82	+ 1.36 ± 4.79	2.12	55	.038	**Significant** (*p* < *.05*)
Nutrition (*n* = 53)	Dietary Habits	41.77 ± 4.53	35.55 ± 3.68	+ 6.23 ± 4.09	11.09	52	<.001	**Highly Significant** (*p* < *.01*)
	Dietary Pattern	99.19 ± 10.38	103.58 ± 9.70	–4.40 ± 8.13	–3.94	52	<.001	**Highly Significant** (*p* < *.01*)

Data are expressed as Mean ± Standard Deviation. Independent paired-samples t-tests were used to compare baseline and endline scores within each group

For Dietary Habits, lower scores indicate improvement (reduction in unhealthy eating behaviors). For Dietary Pattern, higher scores indicate improvement

*NS* Not Significant

*p* <.05 denotes statistically significant differences

*p* <.01 denotes highly significant differences

### Hierarchical multiple linear regression predicting treatment response

Hierarchical multiple linear regression analysis was conducted to identify predictors of treatment response among NAFLD patients. Model 1, which included demographic variables, explained 10% of the variance (F(5,156) = 3.49, *p* = 0.005), with education level emerging as a significant predictor (β = 0.19, *p* < 0.05). Model 2 incorporated baseline clinical variables and increased the explained variance to 32% (ΔR^2^ = 0.22, *p* < 0.001), with baseline weight, ALT, and triglycerides demonstrating significant predictive associations (*p* < 0.01). In Model 3, the inclusion of intervention-related variables increased the explained variance to 61% (F(18,143) = 14.91, *p* < 0.001; Adj R^2^ = 0.57). Significant predictors included nutrition education (β = 0.428, *p* < 0.001), omega-3 supplementation (β = 0.312, *p* < 0.001), physical activity change (β = 0.284, *p* < 0.001), and intervention compliance (β = 0.258, *p* = 0.001). Model diagnostics indicated acceptable model assumptions, including absence of multicollinearity (VIF < 3), normally distributed residuals, and homoscedasticity. For more details, refer to Table 6.

**Table 6:** Hierarchical multiple linear regression models predicting treatment response (*N* = 162)

Model/Variable	Statistic/Estimate (95% CI)	β/r^2^	*p*-value	Interpretation/Note
A. Dependent Variable	Treatment Response Score = Sum of 5 criteria (0–5): Weight loss ≥ 5%, ALT ↓ ≥ 20%, AST ↓ ≥ 20%, TG ↓ ≥ 20%, LDL-C ↓ ≥ 10%. Higher = better response	—	—	Composite index for global therapeutic improvement
B. Hierarchical Regression Predictors				
Model 1 – Demographics	F(5,156) = 3.49, *p* = 0.005, R^2^ = 0.10	Adj R^2^ = 0.07	—	Education level significant (β = 0.19, *p* < 0.05)
Model 2 – + Baseline Clinical	F(12,149) = 5.82, *p* < 0.001, R^2^ = 0.32	ΔR^2^ = 0.22	—	Baseline weight, ALT, TG strong predictors (*p* < 0.01)
Model 3 – + Intervention & Adherence	F(18,143) = 14.91, *p* < 0.001, R^2^ = 0.61	Adj R^2^ = 0.57	ΔR^2^ = 0.29	Final model explains 61% variance (DW = 1.94)
C. Key Predictors – Final Model (3)				
Nutrition Group (vs Control)	B = 1.156 (0.761–1.551)	β = 0.428	** < 0.001**	Large positive predictor (r^2^ = 0.18)
Omega-3 Group (vs Control)	0.842 (0.458–1.226)	0.312	** < 0.001**	Medium–large effect (r^2^ = 0.10)
Physical Activity Change (MET-min)	0.0008 (0.0004–0.0012)	0.284	** < 0.001**	Medium effect (r^2^ = 0.08)
Intervention Compliance (%)	0.024 (0.010–0.038)	0.258	**0.001**	Medium effect (r^2^ = 0.07)
Baseline ALT (U/L)	0.029 (0.009–0.049)	0.217	**0.004**	Small–medium effect (r^2^ = 0.05)
Dietary Pattern Change (score)	0.032 (0.010–0.054)	0.192	**0.005**	Small–medium effect (r^2^ = 0.04)
Baseline Triglycerides (mg/dL)	0.004 (0.001–0.007)	0.186	**0.009**	Small–medium effect (r^2^ = 0.04)
Attendance at Education Sessions (n)	0.068 (0.021–0.115)	0.186	**0.005**	Small–medium effect (r^2^ = 0.04)
Baseline Weight (kg)	0.018 (0.004–0.032)	0.176	**0.013**	Small effect (r^2^ = 0.03)
Education Level (years)	0.042 (− 0.009 to 0.093)	0.095	0.107	NS
D. Model Fit & Diagnostics				
R/R^2^/Adj R^2^ (Model 3)	0.781/0.610/0.569	—	—	High explained variance (57%)
F Change (ΔR^2^)	17.48 (6,143)	—	** < 0.001**	Significant improvement vs Model 2
Durbin–Watson	1.94	—	—	Residual independence satisfied
Shapiro–Wilk	W = 0.986	—	0.124	Residuals normal
Levene’s Test	F = 1.42	—	0.234	Homoscedastic
E. Multicollinearity Diagnostics (Model 3)				
Tolerance = 0.39–0.81	VIF = 1.2–2.5	—	—	No multicollinearity (VIF < 3)
F. ANOVA Summary				
Model 1: F(5,156) = 3.49, *p* = 0.005	Model 2: F(12,149) = 5.87, *p* < 0.001	Model 3: F(18,143) = 14.91, *p* < 0.001	—	Sequential improvement in fit
G. Overall Interpretation	Nutrition and Omega-3 interventions, along with better compliance, higher physical activity, and improved diet scores, significantly predicted treatment response. Baseline ALT and TG remained significant biochemical predictors. Demographic variables showed minimal influence. Final model explained 61% of variance with no violation of assumptions	—	—	Comprehensive predictive model of NAFLD treatment success

All values represent Pearson correlation coefficients

Some variables had slightly fewer cases due to missing responses (e.g., Nature of work)

*p* <.05 = significant (*)

*p* <.01 = highly significant (**)

### Correlation between dietary habits, dietary pattern, and demographic/clinical variables

The results showed several significant associations between dietary behaviors and demographic or clinical characteristics among NAFLD patients. Dietary habits were positively correlated with smoking status (*r* = 0.16, *p* < 0.05) but negatively correlated with family income (*r* = − 0.18, *p* < 0.05), suggesting that healthier dietary habits were more common among non-smokers and those with lower income levels. Dietary pattern was not significantly associated with most demographic variables. Among the background factors, education level showed a strong positive correlation with family income (*r* = 0.42, *p* < 0.01) and a negative relationship with age (*r* = − 0.21, *p* < 0.01) and gender (*r* = − 0.19, *p* < 0.05), indicating that younger and male participants tended to have higher educational attainment. Medication use strongly correlated with age (*r* = 0.50, *p* < 0.01) and past medical history (*r* = 0.73, *p* < 0.01), confirming that older individuals with comorbidities were more likely to be on medication (Table 7).

**Table 7:** Pearson correlation matrix between dietary habits, dietary pattern, and demographic/clinical variables (*N* = 162)

Variables	1	2	3	4	5	6	7	8	9	10	11	12	13	14
1. Dietary Habits	1													
2. Dietary Pattern	−.10	1												
3. Age	−.12	.04	1											
4. Gender	−.06	.01	.00	1										
5. Marital status	−.07	−.05	.07	−.11	1									
6. Religion	.09	−.04	−.04	.03	−.17*	1								
7. Level of education	−.02	.14	−.21**	−.19*	−.03	−.10	1							
8. Nature of work	−.06	.05	.24*	.40**	−.22*	.10	.14	1						
9. Residential area	.11	−.08	−.18*	−.01	−.09	−.04	−.15	−.10	1					
10. Family income	−.18*	.06	−.02	−.19*	.05	−.04	.42**	−.05	−.11	1				
11. Medication in use	−.15	−.03	.50**	.14	.02	.06	−.21**	.11	−.10	−.01	1			
12. Food allergy	−.05	.03	−.00	.09	−.01	.01	−.07	.10	.03	−.09	−.10	1		
13. Smoking status	.16*	.03	.12	.27**	−.27**	.04	−.13	.06	.07	−.13	.01	−.03	1	
14. Past medical history	−.03	.02	.20*	.16*	−.02	−.01	−.05	.22*	−.12	.02	.73**	−.24**	.09	1

All values represent Pearson correlation coefficients

*p* <.05

### Clinical decision support framework for post-intervention NAFLD management

The results showed that post-intervention patients were distributed across five clinical risk categories. About 10.5% were critical, showing persistently high liver enzymes and triglycerides with poor adherence, requiring specialist referral and intensive follow-up. The high-risk group (17.9%) had moderate enzyme elevation and partial adherence, needing structured counseling and monthly reviews. Around 24.1% were moderate-high, showing enzyme improvement and moderate adherence, suitable for quarterly monitoring. The moderate group (31.5%) exhibited normalized enzymes and ≥ 5% weight loss, indicating readiness for maintenance-phase care. Finally, 16% achieved low-risk recovery, with normal biochemical levels and excellent adherence, requiring only annual follow-up and preventive counseling. For more details, refer to Table 8.

**Table 8:** Clinical decision support framework for post-intervention management of NAFLD patients based on combined biochemical, anthropometric, and lifestyle indicators (*N* = 162)

Risk Category	Criteria (Based on Post-Treatment Indicators)	n (%)	Clinical & Biochemical Profile	Key Predictive Indicators	Recommended Clinical Action Plan
Critical (High Risk)	ALT > 45 U/L or AST > 40 U/L + BMI > 32 kg/m^2^ + Triglycerides > 200 mg/dL + no ≥ 5% weight loss	~ 17 (10.5%)	ALT = 54 ± 8 U/L; TG = 236 ± 21 mg/dL; BMI = 34.8 ± 3.2 kg/m^2^	Persistent Grade II steatosis; poor adherence (< 70%); minimal physical activity	Intensify pharmacologic/lifestyle management + specialist hepatology referral + dietitian follow-up every 2 weeks
High	ALT 35–45 U/L or AST 30–40 U/L + TG > 180 mg/dL + weight loss < 3% + moderate adherence (70–80%)	~ 29 (17.9%)	Moderate enzyme reduction; TG = 198 ± 18 mg/dL; BMI = 32.1 ± 2.9 kg/m^2^	Grade I–II steatosis; partial adherence to omega-3 or sessions	Structured counseling + reinforcement of adherence + monthly biochemical review
Moderate-High	ALT < 35 U/L + TG 160–180 mg/dL + weight loss 3–5% + physical activity > 1,800 MET-min/week	~ 39 (24.1%)	TG = 171 ± 14 mg/dL; LDL = 99 ± 17 mg/dL; BMI = 31.0 ± 2.7 kg/m^2^	Improved liver grade; moderate adherence (≥ 80%) + dietary improvement	Maintain current program + quarterly monitoring + peer-based lifestyle support
Moderate	ALT < 30 U/L + TG < 160 mg/dL + weight loss ≥ 5% + high adherence (≥ 90%)	~ 51 (31.5%)	ALT = 26 ± 6 U/L; TG = 149 ± 13 mg/dL; BMI = 29.8 ± 2.4 kg/m^2^	Normalizing liver function; improved ultrasound grade	Transition to maintenance phase + 3-month follow-ups + reinforcement of self-monitoring
Low (Recovered/Stable)	ALT ≤ 25 U/L + TG < 150 mg/dL + BMI < 30 kg/m^2^ + ≥ 10% weight loss + Grade 0–I liver ultrasound	~ 26 (16.0%)	ALT = 22 ± 4 U/L; TG = 132 ± 11 mg/dL; BMI = 28.4 ± 2.2 kg/m^2^	Complete steatosis resolution; excellent adherence; normal lipid profile	Annual follow-up + preventive lifestyle counseling + relapse-risk screening

### Safety and adverse events

Both interventions demonstrated excellent safety profiles. In the omega-3 group (*n* = 56), mild adverse events occurred in 10 participants (17.9%): fishy aftertaste (*n* = 6, 10.7%) resolved with capsule refrigeration, gastrointestinal discomfort (*n* = 4, 7.1%) managed with meal timing, and 2 participants (3.6%) discontinued due to persistent symptoms. No serious adverse events occurred. The nutrition education (*n* = 53) and control (*n* = 53) groups reported no intervention-related adverse events. For more details, refer to Table 9.

**Table 9:** Safety and adverse events (*N* = 162)

Study Group	Adverse Event Type	n (%)	Clinical Presentation	Management & Resolution	Severity & Causality
Omega-3 Supplementation (*n* = 56)	Gastrointestinal discomfort	4 (7.1%)	Nausea, bloating, mild abdominal discomfort	Self-limiting; resolved with dose timing adjustment (taken with meals)	Mild; Probable relationship
	Fishy aftertaste	6 (10.7%)	Unpleasant taste, mild eructation	Resolved with refrigeration of capsules and consumption with food	Mild; Definite relationship
	Intervention discontinuation	2 (3.6%)	Persistent GI symptoms despite management	Discontinued at week 3 and 5; symptoms resolved within 3 days	Mild; Probable relationship
	Serious adverse events	0 (0.0%)	None reported	—	—
Nutrition Education (*n* = 53)	No adverse events	0 (0.0%)	No intervention-related AEs	—	—
Control (*n* = 53)	No adverse events	0 (0.0%)	No intervention-related AEs	—	—

All events were classified according to CTCAE v5.0 guidelines

*AEs* Adverse events, *GI* Gastrointestinal

## Discussion

The present study examined the association between a nutrition education program and omega-3 supplementation with metabolic and hepatic outcomes among patients with non-alcoholic fatty liver disease. The findings indicated statistically significant improvements in several anthropometric and biochemical parameters among participants receiving the interventions, with larger changes observed in the nutrition education group across multiple outcome measures.

Non-alcoholic fatty liver disease has emerged as one of the most prevalent chronic liver conditions worldwide, affecting approximately one-quarter of the global population and representing a significant public health burden [27, 28]. Despite the growing recognition of NAFLD as a major health concern, effective therapeutic strategies remain limited, and lifestyle modification continues to be the cornerstone of management. However, there is insufficient evidence regarding the comparative effectiveness of different intervention approaches, particularly in the context of Middle Eastern populations where dietary patterns and healthcare practices differ substantially from Western settings. Given the importance of these details, we aimed to evaluate the safety and effectiveness of a combined nutrition education and omega-3 supplementation program on metabolic and hepatic outcomes in NAFLD patients.

The demographic profile of our study participants, predominantly middle-aged adults with an average age in the mid-to-late forties, reflects the typical age distribution of NAFLD patients observed globally [29]. This finding aligns with international epidemiological data indicating that NAFLD prevalence peaks during middle age, when metabolic risk factors and sedentary lifestyles become more pronounced [30, 31]. The slight female predominance in our sample is consistent with some regional studies conducted in Middle Eastern populations, though it contrasts with certain Western studies that report higher prevalence among males [15, 18]. This gender distribution may reflect cultural factors influencing healthcare-seeking behavior or differences in metabolic risk profiles between populations.

The high prevalence of married participants and the moderate income distribution observed across all groups mirrors the socioeconomic characteristics commonly reported in urban healthcare settings in Iraq [32]. The significant differences in educational level, occupation, and residential area among groups, while initially concerning, underscore the heterogeneity of NAFLD patients in real-world clinical settings. The higher proportion of employed individuals and urban residents in the omega-3 group may reflect better access to healthcare resources and nutritional supplements, which is consistent with patterns observed in other developing countries [28]. These demographic variations highlight the importance of tailoring intervention strategies to accommodate diverse socioeconomic backgrounds.

The significant improvements in anthropometric parameters observed in both intervention groups represent a clinically meaningful achievement in NAFLD management. The reduction in body weight and body mass index in the nutrition education group was particularly notable, demonstrating that structured dietary counseling can effectively promote weight loss even without pharmacological intervention [33, 34]. These findings are consistent with numerous international studies that have established weight reduction as a primary therapeutic goal in NAFLD treatment [18, 21]. However, the magnitude of weight loss achieved in our study, though statistically significant, was modest compared to some Western trials, possibly reflecting differences in baseline dietary patterns, cultural food preferences, or intervention intensity and duration.

The marked improvements in lipid profile parameters, particularly in the nutrition education group, represent a critical finding given the strong association between dyslipidemia and NAFLD progression [35, 36]. The significant reductions in triglycerides, total cholesterol, and low-density lipoprotein cholesterol, coupled with increased high-density lipoprotein cholesterol levels, suggest that comprehensive nutrition education can effectively address multiple cardiovascular risk factors simultaneously. These lipid improvements align with findings from international studies examining dietary interventions in metabolic syndrome and NAFLD populations [7, 9]. The relatively modest lipid changes observed in the omega-3 group, despite the well-established lipid-lowering properties of omega-3 fatty acids, may indicate that supplementation alone is insufficient without accompanying behavioral modifications.

The substantial decreases in liver enzyme activities, including aspartate aminotransferase and alanine aminotransferase in both intervention groups, with additional alkaline phosphatase reduction in the nutrition education group, provide compelling evidence of hepatic improvement [4, 10]. These enzyme reductions are particularly significant as they represent direct markers of hepatocellular injury and inflammation [37]. The consistency of these findings with previous research from various countries reinforces the effectiveness of lifestyle interventions in reducing liver inflammation [38]. The superior performance of the nutrition education group in normalizing liver echogenicity on ultrasound, with more than one-quarter of participants achieving normal findings compared to minimal improvement in controls, demonstrates the tangible impact of comprehensive dietary counseling on hepatic steatosis.

The remarkable elevation in physical activity levels among intervention groups, particularly in the nutrition education cohort, reveals an important synergistic effect of structured health education programs [16, 18]. The substantial increase in metabolic equivalent task minutes per week suggests that nutrition counseling successfully motivated broader lifestyle changes beyond dietary modification alone. This finding parallels observations from comprehensive lifestyle intervention studies worldwide, which consistently demonstrate that education-based approaches foster holistic behavior change [39]. The lack of significant difference in physical activity between the omega-3 and nutrition education groups indicates that both interventions positively influenced exercise behavior, though through potentially different mechanisms.

The significant improvements in dietary habits and dietary patterns observed exclusively in the intervention groups confirm the effectiveness of both approaches in modifying eating behaviors. The nutrition education group showed particularly impressive changes, with marked improvements in dietary habit scores and enhanced dietary pattern adherence. These behavioral modifications are crucial for long-term NAFLD management, as sustained dietary changes represent the foundation of disease control and prevention of progression [40]. Similar patterns of dietary improvement have been documented in behavioral intervention studies across diverse populations, though the specific dietary changes may vary according to cultural food preferences and baseline eating patterns [30, 40].

The hierarchical regression analysis provides valuable insights into the multifactorial nature of treatment response in NAFLD patients. The progressive increase in explained variance across models demonstrates that while demographic and clinical factors contribute to outcomes, intervention type and adherence behaviors are the primary drivers of success [41]. The strong predictive value of nutrition education, omega-3 supplementation, physical activity change, and intervention compliance emphasizes the critical importance of behavioral engagement and therapeutic adherence in achieving optimal results. These findings align with implementation science research highlighting adherence as a key determinant of intervention effectiveness across chronic disease management programs [30].

Despite the valuable insights provided by this study, several limitations should be acknowledged. The relatively short intervention duration may not capture the long-term sustainability of behavioral changes or delayed effects on hepatic outcomes. The study was conducted in a specific geographic region with unique dietary and cultural characteristics, which may limit the generalizability of the findings to other populations. Additionally, the short food frequency questionnaire used to assess dietary patterns has not been formally validated in the Kurdish-speaking population, which may introduce measurement error in dietary assessment. The absence of a placebo intervention should also be considered, as it may have influenced participant expectations and response patterns. Furthermore, several outcome measures, particularly dietary habits and physical activity, relied partly on self-reported data, which may be subject to recall or reporting bias. Future research should include longer follow-up periods to assess the durability of interventions and explore cost-effectiveness analyses to inform healthcare policy decisions. Additionally, investigating the mechanisms underlying the stronger observed performance of nutrition education compared with supplementation alone could provide useful insights for optimizing intervention strategies in resource-limited settings.

## Conclusion

The findings of this study suggest that nutrition education and omega-3 supplementation were associated with improvements in hepatic function indicators, lipid profile, and physical activity levels among patients with NAFLD in Erbil City. These results indicate that behaviorally oriented interventions may represent a potentially useful approach for NAFLD management, particularly in resource-limited settings.

## Supplementary Information

Supplementary Material 1.

Supplementary Material 2.

## Data Availability

The data that support the findings of this study are available from the corresponding author upon reasonable request.
