# Supplementary material for: Effectiveness of nutrition education program and omega-3 supplementation on body weight, liver enzyme, lipid profile among non-alcoholic fatty liver disease patients
Source: BMC Nutr. 2026 Apr 16;12:100. doi: 10.1186/s40795-026-01309-0 (PMC13202778; doi:10.1186/s40795-026-01309-0)
Supplement: Supplementary file 2 — Supplementary Material 2. [file 40795_2026_1309_MOESM2_ESM.pdf]

Code #: _____________

Control ☐ **Omega-3** ☐ **Nutrition** ☐

Patient name: **Phone:**

Address: **Today's Date:**

SOCIODEMOGRAPHIC CHARACTERISTICS

1. Year of birth: ___________

2. Gender:☐ Male ☐ Female

3. Marital Status: ☐ Single ☐ Married ☐ Widowed ☐ Divorced

4. Religion: ☐ Muslim ☐ Christian ☐ Yezidi ☐ Others------------

5.Level of education:

☐No formal education

☐ Able to write and read

☐ Primary School

☐ Intermediate School

☐ Secondary school

☐ Diploma(Institute)

☐ Bachelor degrees (college)

☐ Postgraduate (Master or PhD) degrees

6. Occupation:

☐ Unemployed
☐ Employee
☐ Student
☐ Housewife
☐ Retired
☐ Self-business
☐ Worker/hand worker
Others……………………..

7. Nature of Work:

☐ Employed full time ☐ Employed part time

8. Residential area: ☐ Urban ☐ Suburban ☐ Rural

9. Family income:

☐ **Below average** (≤ 500000 ID)

☐ **Average** (>500000-100000 ID)

☐ **Above average** (>1000000 ID)

10. Past Medical History:

☐ Non

☐ Hypertension

☐ Type 2 Diabetes mellitus

☐ PCOS

☐ Dyslipidemia

☐ Hypothyroidism

☐ cardiovascular disease

☐ Others: …………………………………

11. Which medications do you currently take?

☐ Non

☐ Aspirin intake

☐ Statin intake(Exclude)

☐ Insulin intake

☐ Biguanide intake (Metformin)

☐ Others……………………….

12. Do you currently take any supplements:

☐ Yes

☐ No

If yes which supplements, do you take……………………………

13. Smoking status:

Do you currently smoke?

☐ Yes ☐ No

If yes type?

☐ Cigarette ☐ Waterpipe ☐ Vape

Ex-smoker

☐ Yes ☐ No

If yes, when did you stop smoking ……….

14. Do you have any particular food allergies?

☐ Yes ☐ No

If yes please specify it……………………

ANTHROPOMETRIC MEASUREMENT AND BIOCHEMICAL PARAMETER

**Anthropometric measurements**	**Baseline**	**End**
**Hight (cm)**		------------------------
**Weight (kg)**		
**BMI**		
**Waist circumference**		
**Hip circumference**		
**Waist to hip ratio (cm)**		

**Biochemical Parameters:**	**Baseline**	**End**
**Lipid profile**		
Triglyceride		
Cholesterol		
LDL		
HDL		
**Liver enzymes**		
AST		
ALT		
ALP		
**Liver ultrasound grade**		
Grade I		
Grade II		

Lifestyle Questionnaire:

Part I: INTERNATIONAL PHYSICAL ACTIVITY QUESTIONNAIRE (IPAQ)

Types 3: Walking physical activities.

Minute/ days: ……………………………….……….. .

Frequency per week ……………..………………………. .

Type 2: Moderate physical activities (doubles tennis, gardening, bicycling at peace, swimming, hiking).

Type: …………………………………….………

Minute/ days: …………………………………….………

Frequency per week ………………...…………………………

Type 1: Intensive (vigorous) physical activities like (heavy lifting, aerobics (running), or fast bicycling).

Type: …………………………………….………

Minute/ days: ……………………………….………... .

Frequency per week ……………..………………………. .

Part II: Dietary Habits

Code	Items	Never (0wk)	Rarely (1wk)	Sometimes (2-3wk)	Often (4-5wk)	Always (+6)
1	Eating at home					
2	Eating outside					
3	I eat regular meals					
4	Eating Breakfast					
5	Eating fast					
6	Eating between meals					
7	Eating before bedtime					
8	Eating fruit before bedtime					
9	I often feel hungry					
10	Drinking tea directly after meals					
11	I eat fatty food					
12	When I see or smell food that I like, it makes me want to eat.					

Part III: DIETARY PATTERN

A short food frequency questionnaire for assessing dietary intakes of non-alcoholic fatty liver disease patients:

**Food**	**Frequency**				
	**Never**	**Daily**	**weekly**	**2-3/week**	**Monthly**
**Meat**					
Lamb Meat	☐	☐	☐	☐	☐
Beef Meat	☐	☐	☐	☐	☐
**Sea food**					
Fish	☐	☐	☐	☐	☐
Shellfish	☐	☐	☐	☐	☐
**Chicken**	☐	☐	☐	☐	☐
**Eggs**					
Fried	☐	☐	☐	☐	☐
Boiled	☐	☐	☐	☐	☐
Omelet	☐	☐	☐	☐	☐
**Refined grain**					
White Bread	☐	☐	☐	☐	☐
White Rice	☐	☐	☐	☐	☐
**Whole Grain**					
Wheat flour	☐	☐	☐	☐	☐
Brown rice	☐	☐	☐	☐	☐
Bulgur	☐	☐	☐	☐	☐
Oat	☐	☐	☐	☐	☐
**Dairy Product**					
Milk	☐	☐	☐	☐	☐
Yoghurt	☐	☐	☐	☐	☐
Cheese	☐	☐	☐	☐	☐
**Fried food**	☐	☐	☐	☐	☐
**Fast food** (Pizza,	☐	☐	☐	☐	☐

Burger……)					
**Vegetables**	☐	☐	☐	☐	☐
Boiled vegetables(broccoli, zucchini,…….)	☐	☐	☐	☐	☐
Fresh Vegetables(cucumber,...)	☐	☐	☐	☐	☐
**Fruits**	☐	☐	☐	☐	☐
**Cooking fats & oil**					
Olive oil	☐	☐	☐	☐	☐
Sunflower oil	☐	☐	☐	☐	☐
Animal butter	☐	☐	☐	☐	☐
Plant butter	☐	☐	☐	☐	☐
**SWEETS** (Baklava, cakes, Jam,……)	☐	☐	☐	☐	☐
**Legumes** (Beans, chickpea, lentils….)	☐	☐	☐	☐	☐
**BEVERAGES**	☐	☐	☐	☐	☐
Water	☐	☐	☐	☐	☐
Packaged fruit juices	☐	☐	☐	☐	☐
Fresh fruit juices	☐	☐	☐	☐	☐
Sweetened beverages (sweetened tea...)	☐	☐	☐	☐	☐
Fizzy drinks	☐	☐	☐	☐	☐
Alcohol (less than 20 g/day in men and 10 g/day in women).	☐	☐	☐	☐	☐
**NUTS and Seeds**	☐	☐	☐	☐	☐
